# Molecular pathological epidemiology of colorectal cancer in Chinese patients with *KRAS* and *BRAF* mutations

**DOI:** 10.18632/oncotarget.5551

**Published:** 2015-10-22

**Authors:** Wenbin Li, Tian Qiu, Yun Ling, Lei Guo, Lin Li, Jianming Ying

**Affiliations:** ^1^ Department of Pathology, Cancer Hospital, Chinese Academy of Medical Sciences & Peking Union Medical College, Beijing, China

**Keywords:** colorectal cancer, KRAS, BRAF, mutation, molecular pathological epidemiology

## Abstract

An investigation of interactive effects of exogenous and endogenous factors and tumor molecular changes can lead to a better understanding of tumor molecular signatures in colorectal cancer. We here report a molecular pathological epidemiology study in a large cohort of 945 colorectal cancer patients. Mutations of *KRAS* (36.6%) and *BRAF* (3.46%) were nearly mutually exclusive. *KRAS*-mutated tumors were more common in female patients (odds ratio [OR] = 1.68; *P* = 0.0001) and never smokers (OR = 1.60; *P* = 0.001). Whereas *BRAF*-mutated tumors demonstrated no discrepancy in aspects of gender and smoking status compared with wild-type tumors. In addition, tumors with *BRAF* or *KRAS* mutations were in correlation with elevated serum level of carbohydrate antigen (CA19-9) and carcinoma embryonic antigen (CEA) and the combination of serum biomarkers and molecular mutation status may enhance the more precise risk stratification of CRC patients. Further studies are needed to define the mechanism brought about by the aforementioned epidemiologic and clinicopathologic characteristics that may help optimize cancer prevention and precision therapy.

## INTRODUCTION

Colorectal cancer (CRC) is a heterogenous disease evolving from the accumulation of genetic and epigenetic modifications [1–3]. Mutations within the *KRAS* and *BRAF* oncogenes lead to constitutive activation of epidermal growth factor receptor (EGFR) signaling pathway. *KRAS* is mutated in 35%–40% CRC and with more than 95% mutations in codons 12 and 13 [4]. Mutation of *KRAS* oncogene is an early event in development of these cancers, exerting a strong influence on the growth of colonic polyps and early cancers [5]. Robust evidence suggests the predictive value of *KRAS* mutation in metastatic CRC treated with anti-EGFR targeted therapy [6, 7]. However, the biological and functional consequences of *KRAS* mutations at codon 12 may be different from those at codon 13 [8, 9]. It has been suggested that patients whose tumors harbor a *KRAS Gly13Asp* mutation may benefit from anti-EGFR mAb therapy [10–12]. Our previous reports have also demonstrated that *KRAS* codon 12 mutation, but not codon 13 mutation, is associated with more positive lymph nodes and higher pTNM stages in colorectal cancer [13]. On the other hand, *BRAF* c.1799T > A (p.V600E) mutation occurs in less than 10% of patients and are a strong negative prognostic marker [14, 15].

To data, few studies have evaluated the associations of epidemiologic factors and tumor molecular features. Consequently, using collected patient questionnaire data from the database of the Department of Pathology, Cancer Hospital, along with the corresponding *KRAS* and *BRAF* mutational status, we evaluated the associations between tumor molecular and epidemiological features.

## RESULTS

### Epidemiologic characteristics

A total of the 945 cases were analyzed for *KRAS*, *BRAF* gene mutations, MMR status and completed patient questionnaire registration. Of these, 945 (100%) and 924 (97.8%) yielded *KRAS* and *BRAF* mutation status, respectively. There were 346 (36.6%) tumors that had *KRAS* mutations, whereas 32 (3.46%) tumors had a *BRAF* mutation. The distribution and frequencies of the epidemiological characteristics are summarized in Table 1.

**Table 1:** Distributions of patients epidemiological characteristics by *KRAS* and *BRAF* mutation status

Characterics	Mutant *KRAS* (*n* = 346)	Wild-type *KRAS* (*n* = 599)	*P*-value	Mutant *BRAF* (*n* = 32)	Wild-type *BRAF* (*n* = 892)	*P*-value
**Sex**			**0.0001**			0.18
Male	175 (50.6%)	379 (63.3%)		15 (46.9%)	524 (58.7%)	
Female	171 (49.4%)	220 (36.7%)		17 (53.1%)	368 (41.3%)	
**BMI (kg/m^2^)**			0.13			**0.04**
<25	232 (67.1%)	372 (62.1%)		26 (81.3%)	567 (63.6%)	
≥25	114 (32.9%)	227 (37.9%)		6 (18.7%)	325 (36.4%)	
**Smoking status**			**0.001**			0.16
Never	251 (73.4%)	373 (63.2%)		18 (56.3%)	602 (68.1%)	
Former/current	91 (26.6%)	217 (36.8%)		14 (43.7%)	282 (31.9%)	
Missing	4	9		0	8	
**Alcohol intake**			0.09			0.53
Never	249 (73.2%)	405 (68.0%)		24 (75.0%)	617 (69.9%)	
Former/current	91 (26.8%)	191 (32.0%)		8 (25.0%)	266 (30.1%)	
Missing	6	3		0	9	
**Diabetes mellitus**			0.84			0.86
Yes	47 (13.7%)	79 (13.2%)		4 (12.5%)	119 (13.4%)	
No	297 (86.3%)	520 (86.8%)		28 (87.5%)	770 (86.6%)	
Missing	2	0		0	3	
**Hypertension**			0.93			0.38
Yes	88 (26.0%)	152 (25.7%)		6 (18.8%)	227 (25.6%)	
No	251 (74.0%)	439 (74.3%)		26 (81.3%)	659 (74.4%)	
Missing	7	8		0	6	
**Chronic GI conditions**			0.74			0.65
Yes	43 (12.4%)	79 (13.2%)		5 (15.6%)	115 (12.9%)	
No	303 (87.6%)	520 (86.8%)		27 (84.4%)	777 (87.1%)	
**CA19-9 (U/ml)**			**0.0001**			**0.004**
<37	243 (70.2%)	518 (86.5%)		20 (62.5%)	736 (82.5%)	
≥37	103 (29.8%)	81 (13.5%)		12 (37.5%)	156 (17.5%)	
**CEA (ng/ml)**			**0.0001**			**0.02**
<5	167 (48.3%)	408 (68.1%)		13 (40.6%)	549 (61.5%)	
≥5	179 (51.7%)	191 (31.9%)		19 (59.4%)	343 (38.5%)	
**Total cholesterol (mmol/L)**	5.46 ± 0.94	4.53 ± 0.97	0.18†	4.29 ± 0.90	4.89 ± 0.97	0.21†
**HDL-cholesterol (mmol/L)**	1.21 ± 0.41	1.16 ± 0.40	0.06†	1.08 ± 0.31	1.39 ± 0.41	0.17†
**LDL-cholesterol (mmol/L)**	2.73 ± 0.85	2.81 ± 0.75	0.08†	2.71 ± 0.73	2.78 ± 0.86	0.65†
**Triglycerides (mmol/L)**	1.27 ± 0.69	1.36 ± 0.47	0.17†	1.34 ± 0.71	1.32 ± 0.74	0.92†

Abbreviations: MMR = mismatch repair; SD = standard deviation.

† Two-sided Kruskal Wallis test

^‡^Two-sided χ^2^ test with continuity correction

^§^Fischer's exact test

Others are two-sided χ^2^ test

Compared with patients with wild-type *KRAS* tumors, those with mutant *KRAS* tumors were more likely to be female (49.4% *vs* 36.7%; OR = 1.68; 95% CI = 1.29 to 2.20; *P* = 0.0001), to be never smoker (73.4% *vs* 63.2%; OR = 1.60; 95% CI = 1.19 to 2.15; *P* = 0.001), to have elevated CA19–9 serum concentrations (29.8% *vs* 13.5%; OR = 2.71; 95% CI = 1.95 to 3.76; *P* = 0.0001) and to have elevated CEA serum concentrations (51.7% *vs* 31.9%; OR = 2.29; 95% CI = 1.74 to 3.01; *P* = 0.0001). However, there were no significant differences in aspects of age, overweight, alcohol intake, diabetes mellitus, hypertension and chronic GI conditions between mutant *KRAS* and wild-type *KRAS* groups. Moreover, compared with wild-type *KRAS* patients, HDL-cholesterol, LDL-cholesterol and triglycerides were not risk factors for mutant *KRAS* patients.

When compared with those without *BRAF* mutated tumors, patients with *BRAF* mutated tumors were more likely to have elevated CA19-9 serum concentrations (37.5% *vs* 17.5%; OR = 2.83; 95% CI = 1.35 to 5.91; *P* = 0.004) and to have elevated CEA serum concentrations (59.4% *vs* 38.5%; OR = 2.34; 95% CI = 1.14 to 4.79; *P* = 0.02). Patients with *BRAF* mutations were more likely to be less overweight compared with wild-type *BRAF* cases (81.3% *vs* 63.6%; OR = 0.40; 95% CI = 0.16 to 0.99; *P* = 0.04). Moreover, compared with wild-type *BRAF* patients, smoking status, alcohol intake, diabetes mellitus, hypertension and chronic GI conditions were not risk factors for mutant *BRAF* patients.

### Associations between factors and mutation status

**Figure 1:**
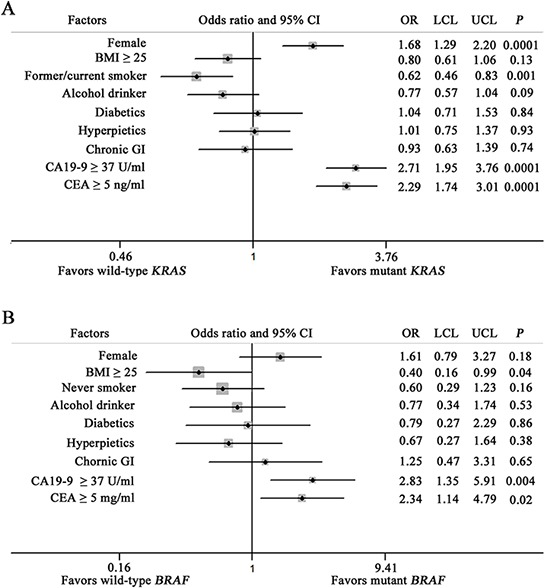
Forest plots of univariate logistic model associations with KRAS A. and BRAFV600E B. mutation status features *P* values are for two-sided Pearson χ2 test. CI = confidence interval; dMMR = deficient mismatch repair; LCL = lower confidence limit; UCL = upper confidence limit; OR = odds ratio.

Univariate logistic regression models identified the following factors as statically significantly associated with having mutant *KRAS* status: female, never smokers as well as elevated CA19–9 and CEA serum concentrations (Figure 1A). *BRAF* mutated tumors were statistically significantly associated with less overweight and elevated CA19–9 and CEA serum concentrations (Figure 1B).

In the analysis using multivariable logistic regression models, we reviewed epidemiological characteristics in Table 2. As shown multivariably, tumors with *KRAS* mutation were statistically associated with female patients, never smokers and elevated serum level of CA19-9 and CEA. In addition, patients with *BRAF*-mutated tumors were statistically significantly to have elevated serum level of CA19-9.

**Table 2:** Multivariate logistic regression model associations between patient, tumor and *KRAS* or *BRAF^V600E^* mutation status

Characteristics	Mutant *KRAS*		Mutant *BRAF^V600E^*	
	OR (95% CI)	*P*	OR (95% CI)	*P*
Female (referent: male)	2.14 (1.07 to 3.86)	0.001	—	—
BMI < 25 (referent: ≥ 25)	—	—	2.35 (0.92 to 3.58)	0.11
Never smoker (referent: Former/current)	1.92 (1.36 to 3.90)	0.004	—	—
CA19-9 ≥ 37 (referent: < 37 U/ml)	3.35 (2.46 to 5.17)	0.001	2.03 (1.04 to 3.98)	0.006
CEA ≥ 5 (referent: < 5 ng/ml)	2.46 (1.55 to 4.60)	0.001	1.52 (0.63 to 2.82)	0.17

CI = confidence interval; pMMR = proficient mismatch repair; OR = odds ratio.

## DISCUSSION

Molecular pathological epidemiology, which was first consolidated by Shuji Ogino and his colleagues, is a relatively new field of epidemiology based on molecular classification of cancer [16]. In recent years, there has been a new direction of this field where we examine an interactive effect of tumor molecular features and lifestyle or other exposure factor on tumor behavior [16–19]. Furthermore, molecular pathological epidemiology has specific strengths on optimizing colorectal cancer prevention and precision therapy. To the best of our knowledge, this is the first study to summarize epidemiologic (ie, cigarette smoking and alcohol drinking) features associated with the *KRAS* and *BRAF^V600E^* mutation status of tumors in a large cohort of Chinese patients.

Cigarette smoking history is a known risk factor for developing colon cancer [20]. Studies have shown that carcinogens found in tobacco smoke can induce cancer-related base substitutions, such as G:C → A:T transitions in *RAS* oncogenes [21]. However, several large studies have indicated that cigarette smoking was more closely associated with incident CRCs characterized by *KRAS* mutation-negative rather than *KRAS* mutation-positive status [22–24]. This is in line with our observations that colorectal cancers from patients with a history of current or former smoking were less likely to harbor a *KRAS* mutation. Although smoking is not associated with the risk for colorectal cancer with *KRAS* oncogene mutations, it may be an early event in the development of colorectal cancers that arise through other underlying genetic pathways, such as mutations in the adenomatous polyposis coli (APC) tumor suppressor gene, P53 over-expression or absence of MLH1 protein expression [25, 26]. Recently studies from the large case-control study suggested that smoking is related to CIMP (CpG Island Methylator Phenotype) and *BRAF* mutations in colon cancer, rather than with microsatellite-unstable cancer [18, 27, 28]. Our study reveals an association between current or former smoking history with the presence of *BRAF* mutations in tumors (43.7% *vs* 31.9%), although this did not reached significantly difference due to small sample size. Tobacco exposure has been shown to stimulate DNA methyltransferase activity that is associated with CIMP and *BRAF* mutations [29, 30]. Previous data indicate that the CIMP-high subgroup, which exhibits a very high frequency of cancer-specific DNA hypermethylation, is strongly associated with epigenetic inactivation of *MLH1* and *BRAF* mutation. [31] Therefore, the *BRAF* mutation can serve as a surrogate marker for the CIMP-high group showing sporadic dMMR status.

To our knowledge, this is the first study to suggest that patients with *BRAF* or *KRAS* mutated tumors were more likely to have an elevated preoperative serum level of CA19–9 and CEA. CA19–9 and CEA are widely accepted tumor serum biomarkers for CRC and elevated preoperative CA19–9 and CEA level have been considered as an independent prognostic factor for DFS (Disease Free Survival) in CRC patients [32]. Recent studies have been suggested that a high preoperative serum CA19-9 level was a significant marker of poor prognosis in patients with all stages of CRC [33]. Strong prognostic effect of *KRAS* and *BRAF* mutations were previously reported by Maughan *et al*. in COIN trial and the OS (Overall Survival) was shorter for patients with any mutation of the two oncogenes compared with all wild-type, irrespective of treatment received [34]. Therefore, tumors with *BRAF* or *KRAS* mutations were in correlation with elevated serum level of tumor biomarkers of CRC and the association of tumor biomarkers and molecular status may indicate the poor prognosis of these patients. Further work needs to estimate the more precise risk stratification of CRC patients based on the combination of serum biomarkers and molecular mutation status.

Our study had several limitations associated with its retrospective nature and single center design. Patients recollected the answers to several questions from memory when filling out the form, hence possibly introducing reporting errors while classifying several patient risk characteristics such as smoking and alcohol history and chronic GI conditions. Moreover, we did not examine rare common mutations in *KRAS* codons 61, 146 and *NRAS* mutations, which seemed to be negative predictive factors to anti-EGFR therapies.

In conclusion, our study suggests that specific epidemiologic characteristics are associated with *KRAS* and *BRAF* mutations in a large cohort of Chinese CRC patients. *KRAS*-mutated tumors are more common in female patients and never smokers. Tumors with *BRAF* or *KRAS* mutations were in correlation with elevated serum level of tumor biomarkers of CRC and the combination of serum biomarkers and molecular mutation status may enhance the more precise risk stratification of CRC patients. Further studies are needed to define the mechanism brought about by the aforementioned epidemiologic and clinicopathologic characteristics that may help optimize cancer prevention and precision therapy.

## MATERIALS AND METHODS

### Study population

The tumor molecular and epidemiological records of 945 patients with corresponding paraffin-embedded material available for molecular analysis were retrospectively collected from the Department of Pathology, Cancer Hospital, Chinese Academy of Medical Sciences, Beijing, China from December 2011 to December 2013. Patients who had a history of preoperative radiochemotherapy or gastrointestinal surgical resection were excluded. The results of pathological characteristics between *KRAS* and *BRAF* mutations of these patients were published in the previous study [13]. The study was approved by the Institute Review Board of the Cancer Hospital, Chinese Academy of Medical Sciences. The methods were carried out in accordance with the approved guidelines. Each participant signed an Institutional Review Board approved informed consent in accordance with current guidelines.

### Risk factor assessment

The following data fields were recorded and included in the analysis: smoking history (never smoker; former smoker: used to smoking but has quit; current: still smoking), alcohol intake (never drink; former: used to drink but has quit; current: still drink), body mass index (BMI, obese: ≥ 30 kg/m^2^; overweight: 25–29.9 kg/m^2^; normal: 18–24.9 kg/m^2^; underweight: < 18 kg/m^2^) and history of chronic gastrointestinal diseases (Crohn's disease, ulcerative colitis or microscopic colitis). All patients had determined serum concentrations of preoperative CEA (Carcinoma embryonic antigen) and CA19–9 (Carbohydrate antigen), which were performed on a Cobas e601 Immunology Analyzer (Roche, Basel, Switzerland). Serum CEA concentrations ≥ 5.0 ng/ml and CA19–9 concentrations ≥ 37 U/ml were regarded as elevated. Serum total cholesterol and triglycerides were quantitatively determined by a colorimetric method and high-density lipoprotein (HDL) and low-density lipoprotein (LDL) cholesterol were determined in a homogenous assay with a colorimetric end point. All measurements were performed on a P8000 Chemistry Analyzer (Roche, Basel, Switzerland).

### *KRAS* and *BRAF* mutation analysis

Assessment of *KRAS* and *BRAF* c.1799T > A (p.V600E) mutational status was performed in the Molecular Pathology Laboratory of Department of Pathology, CICAMS as previously reported [13].

### Statistical analysis

The primary objective of this study was to identify distinct epidemiological features associated with specific *KRAS* and *BRAF^V600E^* mutation status. Differences of patient characteristics and epidemiological factors in the two-dimensional cross-comparison were evaluated statistically by Pearson's χ^2^-test or Fischer's exact test. Statistical tests were two-sided, and *P* < 0.05 were considered significant. Logistic regression models were used to detect associations of these characteristics with each of the specific *KRAS* mutations and provided estimates of odds ratio (ORs) and confidence intervals (CIs). Statistics were carried out using SPSS software (version 16.0 of SPSS, Chicago, IL, USA).

## Abbreviations

CRC: Colorectal cancer
KRAS: kirsten rat sarcoma viral oncogene homolog
IHC: Immunohistochemistry
OR: Odds ratio
CEA: carcinoembryonic antigen
CA 19–9: carbohydrate antigen
CI: confidence interval
EGFR: epidermal growth factor receptor
MMR: mismatch repair
